# Bridging the gap: a mixed-methods real-world pilot of a digital intervention for adults with binge eating

**DOI:** 10.1186/s40337-025-01487-5

**Published:** 2025-12-06

**Authors:** Emma L. Osborne, John Powell, Lee Barker, Catherine Birtwell, Lisa Debrou, Emmanuel Defever, Victoria Francis, Emily Hunter, Nicus Kotze, Amanda Lees, Ciaran Newell, Becca Randell, Claire Rosten, Vivi Yao, Rebecca Murphy

**Affiliations:** 1 Centre for Research on Eating Disorders at Oxford (CREDO), Department of Psychiatry, University of Oxford, Warneford Hospital, Oxford, OX3 7JX UK; 2 Nuffield Department of Primary Care Health Sciences, Radcliffe Primary Care Building, Radcliffe Observatory Quarter, Woodstock Rd, Oxford, OX2 6GG UK; 3 Dorset All Age Eating Disorders Service (DAEDS) - Community Team, Spring Court, Kings Park Hospital, Gloucester Road, Bournemouth, BH7 6JF UK; 4 Credo Therapies, 8 King Edward Street, Oxford, OX1 4HL UK; 5 Innovation Centre, Health Innovation Wessex, Southampton Science Park, 2 Venture Rd, Chilworth, Southampton, SO16 7NP UK; 6 Health Innovation Kent Surrey Sussex, Amelia House, Crescent Road, Worthing, BN11 1RL UK; 7 Health Innovation West of England, Future Space, UWE North Gate, Filton Road, Stoke Gifford, Bristol, BS34 8RB UK

**Keywords:** Binge eating disorder, Bulimia nervosa, CBT-E, Digital intervention, Eating disorders, Enhanced cognitive behaviour therapy, Internet, Online, Smartphone, Website

## Abstract

**Background:**

Many individuals who experience binge eating face significant challenges in accessing timely and adequate treatment, often due to limited healthcare resources. To address this, the digital, programme-led (self-help) version of Enhanced Cognitive Behaviour Therapy (CBT-E) has been developed. This service improvement project piloted the digital programme with adults on a specialist eating disorder service waiting list in the UK’s National Health Service (NHS). Its aim was to assess the feasibility, acceptability, and preliminary clinical effects of a digital programme for adults on a waiting list for an eating disorder characterised by binge eating.

**Methods:**

The digital programme was offered to patients with eating problems characterised by binge eating (binge eating disorder or bulimia nervosa or atypical or subclinical threshold cases), for whom a programme-led treatment was appropriate and who were on a waiting list for a specialist outpatient service. Patients used the programme independently, without any additional support. They completed self-report measures assessing eating disorder features, secondary impairment, and features of depression before and after the programme. Patients provided feedback through semi-structured interviews, and staff completed a survey.

**Results:**

Fifty patients started the programme, and 19 completed all active programme sessions. Those who completed the full programme and the post-programme assessments (*n* = 14) reported significant reductions in binge eating frequency, eating disorder psychopathology, secondary impairment, and features of depression. Qualitative feedback from patients and staff highlighted the programme’s value as a waiting list offer and its role in supporting patients’ progress towards recovery. Some patients expressed a desire for human interaction to help them better engage with the programme.

**Conclusions:**

These findings suggest that the digital, programme-led version of CBT-E is feasible, acceptable, and shows promise in reducing binge eating and related impairments in adults on a waiting list for a specialist outpatient eating disorder services. Offering this evidence-informed programme could help address the challenge of long delays in accessing care. Future research should focus on strategies to enhance patient engagement and adherence, improve human interaction within the programme, and explore ways to scale the intervention to benefit broader populations, including its use as an early intervention.

**Supplementary Information:**

The online version contains supplementary material available at 10.1186/s40337-025-01487-5.

## Abstract (plain language summary)

This project tested the digital self-help version of Enhanced Cognitive Behaviour Therapy (CBT-E) for adults with eating disorders involving binge eating who were on a waiting list for a specialist eating disorder service in the UK’s National Health Service (NHS). Patients used the digital programme on their own, without support from a healthcare professional. Those who completed it reported fewer binge eating episodes, eating disorder symptoms, and feelings of depression. Both patients and NHS staff found the programme helpful for those on the waiting list and useful in supporting recovery. However, some patients felt they would have benefited from more guidance. Overall, the programme was practical, well-received, and led to positive outcomes. The programme could help ease pressure on the NHS while allowing patients to access care more quickly. More research is needed, including a controlled clinical trial, to confirm that the programme is effective and good value for money.

**Supplementary Information:**

The online version contains supplementary material available at 10.1186/s40337-025-01487-5.

## Introduction

### Eating disorders characterised by binge eating

Eating disorders characterised by binge eating, including binge eating disorder (BED), bulimia nervosa (BN), and related conditions, are highly prevalent and contribute to significant physical and psychological impairment, reduced quality of life, and substantial societal costs [3, 4, 25, 26]. These disorders affect approximately 2.8% of women and 1% of men globally for BED and 1.9% of women and 0.6% of men for BN [22].

Despite the severity and prevalence of these disorders, many individuals face substantial barriers to adequate and timely treatment. Shame and stigma, which often surround binge eating [6], can prevent people from seeking help. Furthermore, a shortage of trained therapists and limited availability of specialist services contribute to long waiting lists and difficulties meeting the needs of people seeking help [39]. As a result, only a minority of individuals with eating disorders receive the recommended treatments, even in developed countries [5, 27].

### Challenges in NHS service provision

In the UK, these challenges are exacerbated by underfunding, workforce shortages, and increasing demand within the National Health Service (NHS). Given that timely intervention is essential to improve clinical outcomes [6], prolonged waiting times pose serious concern. Delays in assessment and treatment also result in additional societal costs. A report by *Beat*, the UK’s leading eating disorder charity, found that eating disorders have a significant impact on the whole family, who on average spend over £32,000 on travel to clinics, special food, lost time at work, and other expenses [8]. More generally, Lord Darzi’s independent investigation into the NHS highlighted the need for a significant shift towards technology through digital systems to enhance productivity and patient care [14].

Despite National Institute for Health and Care Excellence (NICE) guidance [35] recommending immediate referral to a community-based, age-appropriate eating disorder service if an eating disorder is suspected, access to treatment within the NHS remains a significant challenge. Many specialist eating disorder services have strict referral criteria and may not accept all cases. There is also often significant variability in service provision for eating disorders characterised by binge eating within the NHS. In some areas, specialist services provide evidence-based interventions, while in others, access is limited or non-existent. People experiencing these disorders often fall through the gaps in service provision.

### Bridging the gap: digital interventions

Digital interventions offer a promising solution to address this treatment gap by increasing accessibility and reach. Programme-led (self-help) interventions deliver treatment through programme content rather than therapist contact [21]. These can be delivered digitally or in print and are particularly well-suited to binge eating presentations as individuals with these conditions respond well to structured, self-guided approaches [16, 38].

Evidence from randomised trials further support the potential of fully self-guided formats. Aardoom et al. [1] found that a web-based, fully automated self-help programme led to significant reductions in eating disorder psychopathology relative to a waitlist control, with no added benefit in treatment effectiveness from therapist support. Similarly, Barakat et al. [7] reported that an online cognitive behaviour therapy (CBT) self-help intervention for BN produced comparable improvements in frequency of objective binge eating across both pure self-help and clinician-supported versions at 24-week follow-up.

As a recent expert consensus statement highlights, “to meet the unprecedented demand for treatment quickly and effectively, it is essential to develop and deliver less resource-intensive interventions that are scientifically supported, accessible, and scalable” ([15] p. 579) and “programme-led and focused interventions are needed to close the demand–capacity gap” (p. 589).

Enhanced cognitive behaviour therapy (CBT-E; [18]) is a leading, evidence-based treatment for eating disorders, endorsed by NICE [35] in both therapist-led and guided self-help forms. While NICE currently recommends guided self-help as the preferred format, given that adherence and outcomes tend to be stronger with guidance [9], evidence indicates that fully self-guided programmes may also be effective for some individuals (e.g., [13, 29, 36]).

A programme-led intervention with no external input offers the most scalable approach, maximising access for those who might otherwise receive no treatment. Given substantial NHS waiting lists for eating disorder services, this approach could empower individuals to engage in structured self-help while awaiting formal treatment. This has the potential to reduce symptom severity before specialist care, shorten time to recovery, and optimise NHS resources. It also aligns with NICE guidance on the need for timely recognition and access to care for eating disorders [35].

### The current project

This service improvement project evaluated the digital, programme-led version of CBT-E and its associated printed programme, *Overcoming Binge Eating* [17, 19]. Designed for scalable use, the programme allows patients to work through it independently or with guidance in the form of non-specialist support. In this project, it was piloted without external support.

This project aimed to evaluate the feasibility of the programme in its broadest sense, with a focus on the workability of its delivery within a routine NHS service. This included the evaluation of its acceptability, considering the perspectives of both staff and patients, as well as completion rates and preliminary clinical outcomes. The patient group were adults with eating disorders characterised by recurrent binge eating who are on an NHS waiting list for a specialist eating disorder service.

## Method

### Design

This was a service improvement project, conducted within an NHS setting, that aimed to improve the quality of care in an existing healthcare service and therefore, in accordance with UK Health Research Authority guidance, formal NHS Research Ethics Committee approval was not required. The project employed a mixed-methods approach, including quantitative self-report measures, semi-structured interviews, and an open-ended survey. Quantitative data were collected through the programme, where patients were provided with information on how their data may be used. Consent was obtained for participation in interviews and surveys, as this was outside of routine care. All data were anonymised, and identifying information was removed in accordance with NHS suppression rules to ensure confidentiality and data protection. All interviews and data analyses were conducted by an independent evaluation service, *Health Innovation Wessex (HIW)*.

### Setting

The *Dorset All Age Community Eating Disorders Service (DAEDS) - Community Team*^1^ provides evidence-based outpatient treatments to individuals in Dorset. As a specialist eating disorder service, it reported a surge in demand due to the COVID-19 pandemic. Patients awaiting assessment are placed on a waiting list and provided with information and self-help tools. On average, the waiting time from referral to being offered the digital programme (where appropriate) was approximately 381 days. In this service improvement project, the programme was offered as a supplementary resource designed to support active waiting rather than to remove patients from the waiting list. Patients were encouraged to engage with the programme while remaining on the waiting list.

### Patients and procedure

*DAEDS* reviewed its waiting list to identify patients who might benefit from the programme at that time in the service. This included adults with BED or BN (or atypical or subclinical threshold cases) who could safely use a self-help programme (e.g., those not engaging in frequent compensatory behaviours, not underweight) and met other criteria deemed important by *DAEDS* (e.g., not severely depressed, pregnant, or engaged in alcohol or substance misuse; see *Suitability and demographic survey* section for further details). Patients who might benefit were contacted by a team member who described the programme and offered participation. The programme was available to patients from October 2023 to May 2024. While completing the programme, none of the patients reached the top of the waiting list for specialist treatment.

### Intervention

The intervention was the digital, programme-led (self-help) version of CBT-E. CBT-E is an empirically supported, therapist-led treatment for eating disorders [18]. The programme is a psychological treatment for people who experience recurrent binge eating. It is an eating-disorder-focused CBT programme delivered via a smartphone application and website. The programme consisted of 12 sessions delivered over approximately 8–12 weeks. Sessions were made available at fixed time intervals, with later sessions spaced further apart. Content was presented primarily in text and figure format and included interactive components such as a real-time self-monitoring tool and task lists. In this service improvement project, the programme was offered as a fully automated (“pure self-help” or “self-guided”) intervention, with no additional guidance or support provided. There was no contact with research or clinical staff during the programme; patients were contacted by the service only at the outset to be invited to take part and again after they stopped using the programme for an assessment call. All other assessments were automated within the programme, and the only researcher contact was for qualitative interviews conducted by *HIW*. Contact was deliberately minimal to reflect real-world conditions under which this intervention is expected to be implemented in routine care.

### Quantitative methods

#### Suitability and demographic survey

After registering for the programme, patients completed the self-report Programme Suitability Questionnaire for this project and provided demographic information, both as part of the digital programme. The clinical service (*DAEDS*) defined the within-programme suitability criteria and thresholds for this pilot project. These were intended to ensure that participation was limited to individuals whose clinical needs could be safely and appropriately met by the programme, based on the other care options available at the time and their particular service pathway. For the purpose of this pilot project, the programme was considered suitable for patients who reported binge eating (≥ 1 objective binge eating episode over the past 28 days) and were able to read and write in English AND the programme was not considered suitable for patients who reported > 56 occasions of self-induced vomiting or laxative misuse over the past 28 days, a body mass index (BMI) < 18.5 or ≥ 50, severe depression (Patient Health Questionnaire-9 [PHQ-9; [28]] score ≥ 20), suicidal ideation or self-harm (PHQ-9 item 9 ≥ 2), pregnancy, or current alcohol or substance misuse.

For individuals for whom the programme was unsuitable, automated feedback explained the reasons and redirected them to alternative support options. These signposting messages were also developed in collaboration with *DAEDS*.

#### Clinical outcomes

Three standardised measures were integrated into the programme at the start (pre-programme) and end (post-programme) to assess:

- Eating disorder features over the past 28 days – assessed using the Eating Disorder Examination–Questionnaire (EDE-Q; [20]), modified for the digital programme (see Supplementary Materials). Two outcome variables were created:
  - A single item assessing the number of objective binge eating episodes (OBEs) experienced. This item aligns with the programme’s focus on reducing binge eating and this item is not included in the global EDE-Q score.
  - The severity of general eating disorder psychopathology, measured by the global EDE-Q score, where 0 indicates the absence of characteristics and 6 indicates the most severe symptoms.
- Secondary impairment due to eating disorder features – assessed using the Clinical Impairment Assessment (CIA; [10]).
- Depressive features – assessed using the PHQ-9 [28].

#### Patient view of progress and satisfaction

In the final session of the programme, patients completed two closed-ended questions about whether their eating problem and their understanding of it had improved. They also provided feedback on the programme through a short survey, which included selected items from the Client Satisfaction Questionnaire adapted for internet-based interventions [11] along with programme-specific questions. Closed-ended items assessed helpfulness, ease of use, likelihood of recommending the programme to others who binge eat, and perceived ability to live the life they wanted after completion. Open-ended questions invited comments on what patients liked most and least about the programme.

#### Data analysis

Descriptive statistics were calculated for the quantitative measures: means and standard deviations for normally distributed data, medians and ranges for non-normally distributed data, and frequencies and percentages for categorical data. Pre-programme and post-programme scores were compared using paired *t*-tests.

### Qualitative methods

#### Data collection

Semi-structured interviews were conducted to collect qualitative data on patients’ experiences with the intervention while waiting for assessment and treatment by *DAEDS*. All patients were given the opportunity to participate in an optional interview conducted by *HIW*. The interview guide, developed collaboratively by *HIW*,* DAEDS*, the *Centre for Research on Eating Disorders at Oxford (CREDO)* at the University of Oxford, and *Credo Therapies*, was informed by the framework for measuring the implementation of behavioural intervention technologies [24], which focuses on key aspects such as acceptability, appropriateness, feasibility, and fidelity. The interviews were conducted via MS Teams or phone to explore patients’ experiences in depth. Interviews (30 to 45 min) were recorded, transcribed, and imported into NVivo 14 for analysis.

Community service staff were also invited to complete a survey about their experiences of offering the intervention to patients on the waiting list and its perceived impact on their roles and the service overall. The survey, developed by *HIW* in collaboration with project stakeholders, collected staff job role information and included ten open-ended questions. These explored opinions of the programme, its suitability as a support option for patients awaiting assessment, perceived benefits for patients, impacts on staff roles and the service, views on offering the programme as guided self-help, comparisons with the book version, and any other reflections. A link to the survey (MS Forms) was distributed to staff by the evaluation leads at *DAEDS*.

#### Data analysis

Data from the interviews and survey were analysed together. Thematic analysis [12] was used to identify themes. After the initial analysis, *HIW* met with CREDO to discuss, refine, and finalise the themes. Free-text survey responses were exported into NVivo 14 and coded using the same framework developed for the patient interview data, due to the high level of consensus on the themes.

## Results

### Suitability and completion rates

During the evaluation period, 91 patients registered for the programme, with 87/91 (96%) completing the Programme Suitability Questionnaire. Based on the criteria defined by *DAEDS*, the programme was deemed suitable for 51/87 (59%) patients. It was considered unsuitable for patients reporting features consistent with severe depression or thoughts of suicide/self-harm (27/36; 75%), a BMI < 18.5 (1/36; 3%) or ≥ 50 (2/36; 6%), those without recurrent binge eating (6/36; 17%), or those reporting features consistent with substance misuse (9/36; 25%).

Among the 51 patients for whom the programme was suitable, one did not complete the pre-programme questionnaires and therefore did not start the programme. Of the remaining 50 patients, 19/50 (38%) completed all active programme sessions (up to and including Session 9), and 14/50 (28%) completed all twelve sessions, including those focusing on maintenance (staying well in the longer term) and the post-programme outcome assessment. The average number of sessions completed was six.

### Pre-programme patient characteristics

#### Demographics and help-seeking

Table 1 presents patients’ demographic information and help-seeking history. Reported barriers to seeking help included shame (26/30; 87%), lack of awareness of effective treatments (22/30; 73%), and uncertainty about who to approach (21/30; 70%). Many had previously attempted to seek help but were discouraged by factors such as long waiting lists (24/28; 86%), being told their problem was not severe enough (11/28; 39%), or the unavailability of local specialists (11/28; 39%).

**Table 1:** Patient demographic, help-seeking, and pre-programme clinical characteristics

Variable	M (SD)/Mdn (IQR)/*n* (%)	Range
Demographics (*n* = 51)		
Age (years)	38.3 (14.0)	21–72
Gender	Female 48 (94%)	–
	Male 3 (6%)	
Ethnicity	White 48 (94%)	–
	Other 3 (6%)	
Help-seeking (*n* = 51)		
Duration of eating problem (years)	18.0 (18.5)	3–59
First time seeking help	Yes 21 (41%)	–
	No 30 (59%)	
Time wanting help (years)	5.0 (13.0)	0.5–54.3
Pre-programme clinical characteristics (*n* = 50)		
Frequency of objective binge eating (past 4 weeks)	12.72 (10.85)	1–56
Eating disorder psychopathology (EDE-Q)	4.06 (0.84)	2.6–5.6
Secondary impairment (CIA)	29.14 (9.48)	14–44
Features of depression (PHQ-9)	12.22 (3.76)	5–22

*M* mean, *SD* standard deviation, *Mdn* median, *IQR* interquartile range. Although the programme was suitable for 51 patients, one patient did not complete the pre-programme assessment and, therefore, could not be included in the analyses. The final sample consisted of 50 patients

#### Eating disorder and mood features

Table 1 shows the pre-programme eating disorder and mood features.

### Clinical outcomes

Patients who completed all 12 sessions and the post-programme assessment (*n* = 14) reported significant reductions in the frequency of objective binge eating, eating disorder psychopathology, secondary impairment, and features of depression (see Table 2). Clinically meaningful changes were observed in the following areas:

- Eating disorder psychopathology: The mean EDE-Q global score decreased to below 1 SD above the community mean (i.e., < 2.77; [32]), indicating a clinically meaningful improvement.
- Secondary impairment: The proportion of patients with a global impairment score below the clinical cutoff of 16 [10] increased by 29% points, from 7% (1/14) at pre-programme to 36% (5/14) post-programme.
- Features of depression: The average score decreased to below the clinical cutoff of 10 [34], reflecting a meaningful reduction in symptoms.

**Table 2:** Comparison of pre-programme and post-programme scores for individuals completing all 12 sessions and the post-programme assessment (*n* = 14)

	Pre-programme M (SD)	Post-programme M (SD)	t [95% CI]	*p*
Frequency of objective binge eating (over the past four weeks)	12.86 (*SD*)	6.07 (*SD*)	−3.7 [−11.05, −2.52]	0.004
Eating disorder psychopathology (EDE-Q)	3.69 (*SD*)	2.72 (*SD*)	−1.88 [−1.46, −0.47]	< 0.001
Secondary impairment (CIA)	27.29 (*SD*)	21.21 (*SD*)	−2.27 [−11.84, −0.29]	0.041
Features of depression (PHQ-9)	12.36 (*SD*)	8.14 (*SD*)	−3.40 [−6.89, −1.53]	0.005

### Waiting list changes

Patients invited to register for the programme were informed at the point of registration that using the programme would not affect their access to further specialist treatment from the service, should they need it. *DAEDS* offered a review call to each patient who completed the programme. By May 2024—a later time point than when post-programme assessment data were collected—*DAEDS* had reported outcomes for 15 patients (compared with 14 at the time of data collection). Of these, six (6/15; 40%) were discharged from the waiting list (three had no further need for support and three could not be contacted), two (2/15; 13%) remained open to the service following referral to other services for co-existing conditions, and seven (7/15; 47%) were offered further treatment by *DAEDS*.

### Perceived improvement in eating problem and understanding

When asked about the current state of their eating problem, most patients (12/14; 86%) reported that it was either “much better” (4/14; 29%) or “somewhat better” (8/14; 57%). Similarly, the majority (11/14; 79%) indicated that their understanding of their eating problem had improved, describing it as “much better” (3/14; 21%) or “somewhat better” (8/14; 57%).

### Patient satisfaction

All patients found the programme helpful, with 3/13 (23%) rating it as “very helpful” and 10/13 (77%) as “somewhat” or “moderately” helpful. All also found the programme easy to use, with 7/13 (54%) rating it as “very easy to use” and 6/13 (46%) finding it “moderately” or “somewhat” easy to use. When asked whether they would recommend the programme to someone who binge eats, patients gave a mean score of 6.6 (*SD* = 2.1) on a 1 (not at all likely) to 10 (extremely likely) scale. Notably, most patients (8/13; 62%) reported feeling more able to live the life they want after completing the programme.

In response to open-ended questions, patients valued the programme for its privacy and structured and achievable steps. However, some found the programme insufficiently tailored to their needs, either too fast-paced or too short in duration, and lacking a channel for communication.

### Qualitative outcomes

**Fig. 1:**
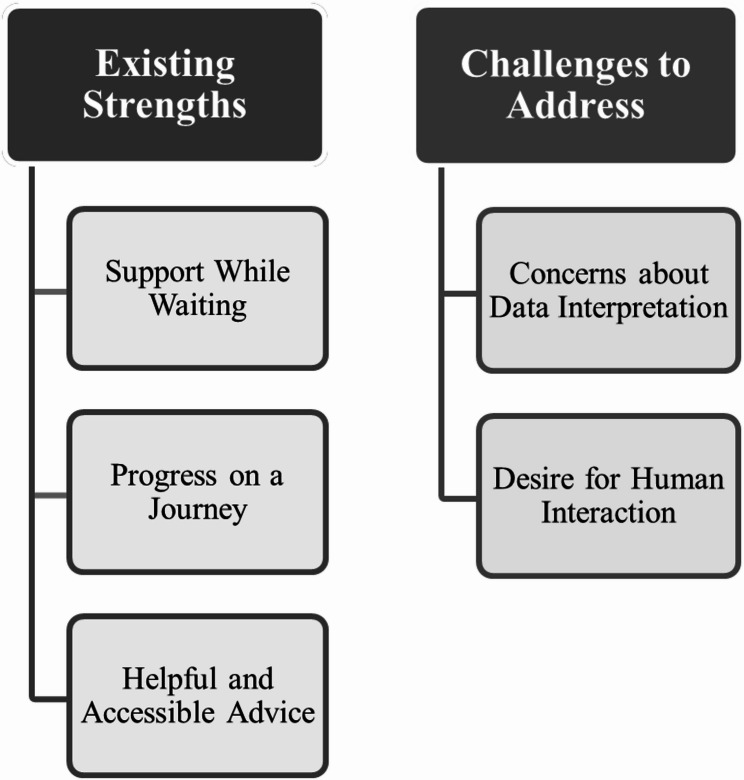
Thematic map

Four patients who had completed or nearly completed the programme were interviewed and eight staff completed the survey (8/67, 12%). Staff included a CBT therapist, community services manager, research assistant, administrator, consultant psychiatrist, eating disorder specialist practitioner, and mental health support worker. In collaboration with CREDO, feedback was organised into two themes: Existing Strengths and Challenges to Address (see Fig. 1).

### Existing strengths

#### Support while waiting

Patients consistently emphasised the value of the digital programme during the waiting period. One patient expressed relief at not being *“forgotten”* (Patient 1) and appreciated the service’s effort to stay engaged. They elaborated: *“To have some support whilst you wait is quite unusual*,* and I was really grateful for that […] it really did make my views of the service change”* (Patient 1). They also highlighted how the programme provided a sense of focus during a challenging time. Another patient described the programme as enhancing their self-help efforts, saying, *“I was very excited […] to kind of assess where I am*,* try and get a real handle on it”* (Patient 4).

Staff echoed these sentiments, recognising the programme as a *“a good intervention for people who cannot access other treatments due to long waiting times or lack of provision in their local area”* (Staff 2). It was described as *“a valuable resource for people”*, especially given that *“our waiting list is two years”* (Staff 4).

#### Progress on a journey

The programme was consistently reported to play a role in patients’ recovery journeys, although the nature of this role varied. One patient stated it *“helped me to begin my progress*,* my recovery”* (Patient 2). Staff also recognised its value early in recovery, seeing it as a preparatory tool that could increase readiness for therapy, such that individuals receiving further professional support might only require *“a lower intensity of therapy”* (Staff 2). Some staff even suggested offering the programme before referral to specialist services, with one remarking, *“It would be useful being given to patients by GP before possible input by an eating disorder service or to reduce the need to have to go to an eating disorder service”* (Staff 5).

In contrast, another patient saw the programme as a way to consolidate prior self-help efforts, explaining it helped them *“assess where I am and […] maybe take myself off that waiting list”* (Patient 4). Some staff viewed the programme as potentially sufficient on its own, with one saying, *“[It] sets patients up well to do treatment in the service OR it is enough…and they feel they don’t need the support of the service anymore”* (Staff 1). This suggests the programme could reduce demand for assessments and treatment.

#### Helpful and accessible advice

Both patients and staff found the programme accessible. Patients appreciated that *“it’s just easier to have everything on your phone”* (Patient 4), while staff noted it *“might be more engaging than the Overcoming Binge Eating book resource we previously recommended”* (Staff 2). The programme’s *“easy-to-use”* design (Patient 1) and presentation of information in *“lovely bite-sized pieces… not overwhelming to the eye or to the mind”* (Patient 2) were particularly valued.

Patients also found the content helpful, especially the guidance on regular eating, which one described as *“quite liberating”* after previously considering eating between meals a *“no-no”* (Patient 3). This guidance helped patients become *“more mindful about the need to eat regularly”* and to *“align [themselves] and be in a good place”* (Patient 4). Similarly, patients noted reduced binge eating and an improved ability to resist urges and manage setbacks. As one patient put it, *“That’s kind of helped me just think*,* OK*,* I’ve messed up*,* but I can still retrieve things […]. Whereas before*,* I thought… I’ve messed up*,* so I’m not going to bother for the day—just keep binge eating”* (Patient 2). Staff also observed positive changes in patients’ symptoms.

### Challenges to address

#### Concerns about data interpretation

While patients recognised the programme’s strengths, some aspects caused uncertainty. One patient questioned who could access the information they entered and how their progress would be assessed: *“If I’m entering all this information*,* although it’s helping me*,* how is anybody going to know? Is there anybody that looks at this information […] Who? What’s happening to this information…how does anybody know whether we’re progressing or not?”* (Patient 2).

Another patient wondered how their progress would be interpreted after being unwell for a few days. They felt it would have been helpful *“to actually have been able to say the reason”* (Patient 3) they had not followed the programme. They explained, *“I was off my food and not very well rather than… wasn’t actually bothering”*, and expressed concern that *“whoever’s analysing the data won’t know”* (Patient 3).

This feedback highlights an opportunity to enhance transparency about how patient data is used and progress is assessed, as well as to provide options for patients to communicate context around their engagement with the programme.

#### Desire for human interaction

Building on the concerns described above, some patients expressed a desire to discuss practical issues, such as missed sessions, illness, or rescheduling preferences. Without the capacity for two-way communication, one patient felt like *“It’s just*,* it’s nothingness. So*,* there’s a real lack of the human side of it there”* (Patient 4). Patients therefore suggested *“It would have been helpful to have had a chat function or…some way of contacting someone that they could answer back at some point*,* if you had any questions or wanted anything clarified” because “at some point I felt a bit alone and…I wanted some more clarification or just some additional kind of sounding…”* (Patient 1).

While these patients self-selected for the interview and may represent a subset who prefer synchronous communication, their feedback highlights an opportunity to explore improved contact and support for patients working through the programme independently.

Some staff also acknowledged that some patients would benefit from clinician input and emphasised the importance of offering guided self-help (GSH) as well: *“I think it’s good for some patients*,* but I would say that I think it’s just as important to offer GSH by working with patients as some people don’t find online work helpful—so I think we still need to be flexible to offer working with patients*,* but I think adding [the programme] is great to offer to those that it might be suitable for”* (Staff 1).

## Discussion

### Overview of findings

This real-world service improvement project evaluated the feasibility of a novel digital programme for patients with recurrent binge eating, focusing on the workability of delivering it within a routine NHS service to those on a waiting list for specialist assessment and treatment. Preliminary clinical outcomes found reductions in binge eating and related impairments for those who completed it. The programme was valued by both patients and staff, with most patients who completed the full programme reporting a better understanding of their eating problem and feeling more able to live the life they wanted. Qualitative feedback from staff and patients highlighted several strengths of the programme, including its value in providing support while on the waiting list, its role in progressing patients’ recovery journeys, and the helpful and accessible advice it provided. Notably, several patients were discharged from the waiting list after completing the programme, suggesting potential cost savings for the service.

By offering this programme, the NHS was able to provide an evidence-informed option for patients on a waiting list, helping to address the challenge of long delays in accessing care. Importantly, the patients described experiencing multiple barriers to seeking help in the past including shame, uncertainty about where to seek support, and difficulties accessing specialist services. For those who had attempted to seek help, long waiting lists were cited as the most significant obstacle to receiving treatment. This underscores the potential for the programme to bridge this gap by providing a more accessible pathway to care.

This evaluation contributes to the growing body of evidence on digital interventions for eating disorders, particularly in bridging gaps in care for underserved populations. A meta-analysis by Linardon et al. [30] reported moderate to large effects for e-mental health interventions compared with control conditions on eating disorder psychopathology, weight and shape concerns, and dietary restraint, although effects on binge or purge frequency were smaller and nonsignificant. While these findings are drawn from randomised controlled trials, the present evaluation extends this evidence by demonstrating improvements in binge eating and related features within a routine NHS clinical service, supporting the potential value of fully self-guided digital interventions in real-world settings. Previous research has also highlighted the value of web-based, guided self-help programmes in bridging the waiting time for outpatient treatment for bulimic-spectrum disorders [40]. This project builds on those findings by demonstrating the feasibility of a fully self-guided approach, which is essential for scaling up interventions to address the significant treatment gap and limitations of traditional care [27].

The restricted demographic profile of the patient group—predominantly White and female—may reflect the characteristics of the local Dorset population, which is less ethnically diverse than other regions in the UK. Eating disorder services also typically see a majority of female patients. Notably, the mean age was 38 (range 21 to 72), challenging the assumption that digital interventions only suit younger people.

Delivering the programme in a non-research, real-world NHS setting presented some implementation challenges, including differing patient expectations (e.g., individuals on a waiting list may anticipate in-person therapy) and limited access to technical support, which may have contributed to dropout. It is important to note that patients on a specialist eating disorder waiting list are typically expecting to receive treatment from a specialist therapist. Offering a digital programme was an entirely novel approach for the service, which may also have influenced engagement and completion rates. The completion rate in this evaluation (19/50; 38%) aligns with reported averages for e-health interventions targeting eating disorders (e.g., 36%; [30]). Given that this intervention was implemented within a specialist NHS outpatient eating disorder service, where patients typically present with more complex conditions than in the community, this completion rate can be considered adequate. Patients reported a long duration of eating problem (median: 18 years; range: up to 59 years), and pre-programme mean scores indicated clinically severe impairment. These findings highlight the long-standing nature and severity of the difficulties faced by this group, which likely influenced engagement. Importantly, the digital programme demonstrated value to the service, staff, and patients who completed it.

Several key learnings about the future use of the digital, programme-led version of CBT-E emerged from this pilot, which have since been acted upon. The programme was deemed unsuitable for 41% of patients. To enhance accessibility, closer collaboration with services will be undertaken to ensure that programme suitability criteria are carefully assessed. In particular, thresholds related to depressive symptoms will be carefully set at an appropriate level, acknowledging that low mood is common in this patient group. The within-programme suitability screening process has also been updated to be more flexible, allowing services to enable or disable it as needed, based on their capacity to provide their own assessments. In response to patients’ “Desire for Human Interaction”, identified through qualitative analysis, and to better support patients using the programme independently, technical assistance options were introduced, and a structured support process was established. Additional information was also developed to provide patients with details about the programme through a series of frequently asked questions. This was done alongside improvements to the programme itself. These adaptations represent progress in expanding the programme’s accessibility and user engagement.

### Strengths and limitations

A key strength of this project was its relevance and timeliness in addressing a critical gap in healthcare services: the unmet needs of individuals who experience recurrent binge eating and face prolonged NHS waiting times. The digital programme provides a scalable, evidence-informed solution to healthcare resource constraints, demonstrating feasibility and acceptability. Closely derived from a well-established, evidence-based treatment for eating disorders, the programme led to meaningful reductions in binge eating and related impairment for those who completed it. These findings strengthen the evidence base for digital interventions, particularly for underserved populations on waiting lists. The project used a mixed-methods approach, combining quantitative outcome data with qualitative feedback from patients and staff to provide a comprehensive evaluation. Interviews and data analysis were conducted by an independent body, enhancing objectivity and credibility.

The project also had several limitations. As a pilot with a small sample size and post-programme assessment data available for only 14 patients, it was not statistically powered to detect definitive effects. Consequently, the findings should be interpreted cautiously, given their limited generalisability. The evaluation design was also prone to selection bias, as patients may have volunteered due to anticipating personal benefit, making the findings less representative of the entire waiting list population. Additionally, completer bias may have influenced the results, as those who finished the programme were more likely to experience improvements. Moreover, the absence of a control group prevents firm conclusions about whether observed improvements were attributable to the programme or to external factors such as regression to the mean, where symptoms naturally improve over time. Although spontaneous remission is less likely given the programme’s short duration, future studies incorporating a comparison arm is essential to strengthen evidence of effectiveness.

The lack of follow-up data also limits understanding of whether improvements were sustained, underscoring the need for future studies to include follow-up assessments. Finally, qualitative insights were based on a small sample of just four patients, which may not fully capture the range of perspectives. While qualitative research values depth over breadth, a larger sample would allow for a wider range of perspectives and enhance the richness and diversity of the findings.

### Implications for clinical practice

This programme has the potential to offer timely support for patients awaiting specialist assessment and treatment, potentially reducing or eliminating the need for further intervention. This could enable healthcare services to allocate resources more efficiently, ease staff workload, and prioritise complex cases. Patient and staff feedback in this evaluation identified areas for improvement, which, if addressed, could enhance patient adherence and optimise clinical outcomes.

Using this intervention during the waiting period aligns with an evolving perspective of reframing a ‘waiting list’ as a ‘preparatory period’ [15], encouraging patient engagement through patients gaining an understanding of their eating disorder and initiating behavioural change. Early intervention may reduce waiting list size by facilitating early improvement, preventing deterioration, and improving treatment efficiency. Patients achieving progress may not require more treatment or need fewer or lower-intensity sessions, enhancing service capacity. Consistent with this, a clinician involved in the pilot confirmed a reduced waiting list for their service.

Beyond waiting-list interventions, this digital programme could integrate effectively at various care pathway stages. Early implementation in primary care could enhance accessibility, allowing self-referral without delays. It might also serve as a first-line treatment, adjunct to standard care, part of blended care, or a component of maintenance programmes for sustained recovery. Evaluating these potential uses within routine services should be a focus of future research, in line with priorities identified by the Digital Mental Health Question (DigitalMHQ) initiative [31].

### Recommendations for programme improvements and further research

Further controlled research is required to evaluate clinical and cost benefits comprehensively. Improving engagement, adherence, and completion rates is a priority, with patient and staff feedback suggesting the addition of guidance or support to address a desire for human interaction. Although incorporating guidance may enhance adherence [33], scalability could be impacted. Investigating innovative, scalable solutions, such as generative artificial intelligence or large language models, presents a promising avenue [37].

Additionally, more real-world studies are necessary to assess effectiveness across diverse settings, exploring barriers and facilitators for integration into routine care. They can also determine whether the programme can be scaled up and sustained to meet high levels of demand. Encouragingly, this programme has already been adopted by the service following this pilot, demonstrating initial feasibility. Further piloting the programme in services operating in more diverse regions would be valuable to assess whether the intervention can effectively engage individuals from a broader range of ethnic and gender groups.

Moreover, research could examine whether previous use of the programme influences therapist-led treatment experiences, patient confidence, and therapeutic efficiency. Anecdotal feedback from one of our experts by experience suggests patients engaging beforehand may find therapy less daunting and build self-efficacy and confidence in treatment, optimising therapist time and improving outcomes.

Finally, applying the nonadoption, abandonment, scale-up, spread, and sustainability (NASSS) framework [2, 23], which provides a model for understanding the complexities of implementing digital interventions in real-world settings, could inform adaptations and research on its long-term sustainability.

## Conclusion

This evaluation provides preliminary evidence to suggest that the digital, programme-led version of CBT-E may be feasible, acceptable, and clinically beneficial for adults with recurrent binge eating on a waiting list for a specialist eating disorder service. Delivered independently without therapist support, the programme led to improvements in binge eating, general eating disorder psychopathology, related impairment, and depressive features among those who completed it. Both patients and staff valued the programme’s accessibility and its potential as a waiting list intervention, though some patients expressed a desire for human interaction and support. These findings suggest that the programme could help address long NHS waiting times, reducing service demand while offering timely and meaningful support to patients in need.

## Supplementary Information

Supplementary material 1.

## Data Availability

The datasets generated during this project are not publicly available due to privacy protection concerns.

## Footnotes

1 From here on, referred to as *DAEDS*.
