# Supplementary material for: Bridging the gap: a mixed-methods real-world pilot of a digital intervention for adults with binge eating
Source: J Eat Disord. 2025 Dec 6;14:11. doi: 10.1186/s40337-025-01487-5 (PMC12797492; doi:10.1186/s40337-025-01487-5)
Supplement: Supplementary file 1 — Supplementary material 1. [file 40337_2025_1487_MOESM1_ESM.pdf]

Supplementary Materials

EDE-Q Modified for the Digital Programme

The modification to the Eating Disorder Examination–Questionnaire (EDE-Q; Fairburn & Beglin, 2008) in the digital programme does not affect calculation of the EDE-Q global and subscale scores. The wording of questions 13, 14, and 15 in the EDE-Q in the digital programme have been modified. The reason for this is to include a measure of subjective binge eating, which refers to episodes of loss of control over eating when the amount of food consumed is not objectively large. The original EDE-Q does not tell us about subjective binge eating; however, the modifications to questions 13–15 tell us about frequency of subjective binge eating without additional burden.
